# Raspberry, Rape, Thyme, Sunflower and Mint Honeys Authentication Using Voltammetric Tongue

**DOI:** 10.3390/s20092565

**Published:** 2020-04-30

**Authors:** Daniela Pauliuc, Florina Dranca, Mircea Oroian

**Affiliations:** Faculty of Food Engineering, Stefan cel Mare University of Suceava, 720229 Suceava, Romania; daniela.pauliuc@fia.usv.ro (D.P.); florina.dranca@usm.ro (F.D.)

**Keywords:** honey, melissopalynological analysis, VE tongue, authentication

## Abstract

The aim of this study was to authenticate five types of Romanian honey (raspberry, rape, thyme, sunflower and mint) using a voltammetric tongue (VE tongue) technique. For the electronic tongue system, six electrodes (silver, gold, platinum, glass, zinc oxide and titanium dioxide) were used. The results of the melissopalynological analysis were supplemented by the data obtained with the electronic voltammetric tongue system. The results were interpreted by means of principal component analysis (PCA) and linear discriminant analysis (LDA). In this way, the usefulness of the working electrodes was compared for determining the botanical origin of the honey samples. The electrodes of titanium dioxide, zinc oxide, and silver were more useful, as the results obtained with these electrodes showed that it was achieved a better classification of honey according to its botanical origin. The comparison of the results of the electronic voltammetric tongue technique with those obtained by melissopalynological analysis showed that the technique was able to accurately classify 92.7% of the original grouped cases. The similarity of results confirmed the ability of the electronic voltammetric tongue technique to perform a rapid characterization of honey samples, which complements its advantages of being an easy-to-use and cheap method of analysis.

## 1. Introduction

Honey is a semi-liquid substance that contains pollen, wax, pigments [1], and was characterized in terms of physicochemical components as a mixture of sugars, minerals, proteins, amino acids, enzymes, volatile substances, phenolic acids, flavonoids and vitamins [2]. As it is considered the only natural sweetener used without processing, honey is often consumed both for its nutritional value, taste and aroma but also for its beneficial effects on human health [3,4,5].

Monofloral honey has a refined and unique aroma and taste, which are factors that contribute to considering this honey type a high-quality product. Thus, monofloral honey is the product most susceptible to being falsified by incorrect labeling and by mixing with lower quality honey [6].

The validation of the authenticity of honey is related to the identification of the botanical and geographical source. The botanical source of honey is closely related to its price, and therefore, to increase their profit, producers have the tendency to adulterate it. The determination of the geographical origin is an important parameter in terms of honey differentiation and value. Depending on the geographical origin, the environment and the area where the honey bees are located, honey can acquire different characteristics and properties [4].

Honey can be classified as “monofloral” or “polyfloral” depending on the dominant pollen, where the first term refers to honey coming from a single plant and the latter to the presence of no type of dominant pollen in the sample. The method that is usually applied in order to identify the predominant pollen grains is melissopalynological analysis [7,8]. Analytical and quantitative methods, namely, high performance liquid chromatography (HPLC) [9] and other instrumental methods such as spectrophotometry, mass spectrometry and emission spectroscopy [10], capillary electrophoresis [11,12], gas chromatography [13], atomic absorption spectroscopy (AAS) [14], layer chromatography (TLC) [15], isotope ratio mass spectrometry (IRMS) [16], near-infrared (NIR) spectroscopy [17] and nuclear magnetic resonance (NMR) spectroscopy [18] are often used for botanical authentication of honey. Subsequently, since all analytical methods are time consuming, involve complex sample preparation and require trained personnel, many researchers aimed to identify the type of honey by electrochemical analysis, using an electronic tongue [19,20].

The electronic tongue is a sensor system that is combined with model recognition tools and has also been used in the qualitative analysis of complex liquid media, such as food and beverages [21]. This system of analysis was proven effective because it has the ability to distinguish complex, even similar liquids [22]. This set of non-specific sensors does not obtain information about the nature of the compounds examined as traditional analytical methods do but presents the fingerprint of the food material [23]. Compared to traditional analytical methods, the electronic tongue presents a number of advantages as it is a rapid measurement technique and contains easy-to-use tools to evaluate the quality of food [24]. Besides the speed of the method, the electronic tongue system also presents a low price for analyzing liquids, such as beer, red wine, tea, juice, vegetable oil and mineral water [25].

In the electronic tongue system, several measurement methodologies can be used to address the detection elements, the most used being the electrochemical ones, namely, potentiometry, amperometry and cyclic voltammetry. An important contribution to this method was made by Riul et al. [26] who introduced the use of small impedance measurements in electronic tongue. Low signal impedance measurements offer the advantage of using materials that make up the detection units that do not have to be electroactive and do not need a reference electrode [26].

Some researchers have used the electronic tongue system to classify honey samples according to their botanical or geographical source [27,28], and others such as Escriche et al. [29] performed a qualitive analysis of honey qualitatively using five electrodes (Ag, Ni, Co, Cu and Au) to determine only the total antioxidant capacity of samples.

Voltammetry has many advantages such as simplicity, high sensitivity, versatility, good noise ratio and robustness [30]. In this technique, a potential is applied to the working electrode; the active redox species are reduced or oxidized on the electrode surface, and the information is obtained from the transient current [31]. The electronic tongue instrumentation has among its components a series of detection units that can be composed of ion selective electrodes, lipid membranes, conductive polymers or noble metals [24]. In an electronic tongue system, the working electrodes are the voltammetric sensors that provide data (voltammograms) about the redox processes that are carried out in the solution when a potential is applied, processes that correspond to the electroactivematerial chosen [28].

The development of harmonized analytical methods is supported by the European Community in order to verify the compliance with the quality specifications for different types of honey. Therefore, the fast, cheap and reliable tool that can be implemented in a handheld instrument used in the field and which can confirm that a particular sample belongs to a floral species is the electronic tongue. Moreover, the electronic tongue helps the marketing chain to detect the adulteration of honey. This method tends to be a reference tool in scientific research and not necessarily a method usually used for identifying the types of honey available on a market [32,33].

The purpose of this paper was to validate the results obtained with a voltammetric electronic tongue for honey samples from Romania with those provided by melissopalynological analysis.

## 2. Materials and Methods

### 2.1. Materials

A total of 41 monofloral Romanian honey samples from different botanical origin (10 samples of mint, 9 samples of raspberry, 4 samples of thyme, 8 samples of sunflower and 10 samples of rape honey) were used for analysis. The samples were from the honey production of 2017 and 2018.

### 2.2. Methods

#### 2.2.1. Melissopalynological Analysis

Melissopalynological analysis was performed following the method of Louveaux, Maurizio and Vorwohl [34]. From each honey sample, 10 g were weighed and mixed with distilled water (40 mL), and then, the samples were centrifuged at 4500 rpm for 15 min. The residue resulting from centrifugation was again mixed with water and centrifuged for another 15 min.

The sediment resulting from the second centrifugation was spread on a microscopic lamella and analyzed under a microscope (Motic, Xiamen, China) at ×40 magnification.

#### 2.2.2. Electrochemical Measurement

Prior to the start of the experiment, the honey samples were liquefied at a temperature of 50 °C.Then, the solution for analysis was prepared as follows: 8 g of honey was weighed, transferred into a 50 mL volumetric flask and filled to the mark with deionized water.

The measuring system consisted of a PGSTAT 204 with FRA32M module (Metrohm, Filderstadt, Germany) coupled to electrodes. For the electrochemical measurement, rod type electrodes were used, namely, a reference electrode (Ag/AgCl), a counting electrode (Glassy Carbon Electrode Rod) and working electrodes (a glass electrode, Au, Ag, Pt, ZnO and TiO_2_) (Metrohm, Filderstadt, Germany). The silver electrode (6.1204.130 Ag, Methrom, Filderstadt, Germany), platinum electrode (6.1204.120 Pt Methrom, Filderstadt, Germany), gold electrode (6.1204.140 Au, Methrom, Filderstadt, Germany) and glass (6.1204.600 GC, Methrom, Filderstadt, Germany) were 2 mm in diameter and zinc oxide, and titanium dioxide electrodes were 5 mm in diameter.

The following chemicals were used for electrodes: 99% purity graphite (Carl ROTH, Karlsruhe, Germany), 99.0–100.5% purity titanium dioxide (Sigma-Aldrich, Hamburg, Germany), 99.9% purity zinc oxide (Sigma-Alorich, Hamburg, Germany) and paraffin oil 99.9% (Tis Farmaceutic S.A., Bucharest, Romania).

The working electrodes of Ag, Pt and Au were purchased and used as such, while those of ZnO and TiO_2_ were manually built in the laboratory using the method proposed by Tiwari et al. [28], as follows: 0.7 g of graphite was mixed with 0.3 g of paraffin oil, and when the mixture became homogeneous, 0.1 g of ZnO was added for the first electrode and TiO_2_ for the second, respectively. The empty electrodes were filled with these mixtures and left to dry for 24 h.

The metal electrodes of Au, Ag and Pt were used because they are the most common metal electrodes in e-tongue systems and are noble metals with very good conductivity, while the glass electrodes have a good conductivity and a low cost. The addition of different oxides into graphite increases the conductivity, and they are low cost.

The technique used for analysis was potentiostatic cyclic voltammetry, and NOVA 2.0 software (Metrohm, Filderstadt, Germany) was used to record the experimental data. The voltage was set from −1 V to +1 V, with a scan rate of 0.5 mV/s for electrodes.

Data reading was performed 5 min after immersing the electrodes in the vessel with the honey solution obtained by the method described above. The analysis of the evidence consisted of 1664 readings, which were made in 45 s. Each analysis was performed in duplicate. Between two procedures, the electrodes were rinsed with deionized water and sanded with filter paper.

#### 2.2.3. Statistical Analysis

Principal component analysis (PCA) and linear discriminant analysis (LDA) were used to interpret the results obtained. PCA is used to explain the variation of experimental data (Wold et al., 1987), and this extracts the most important information from the database.

## 3. Results

### 3.1. Melissopalynological Analysis

The melissopalynological study is recognized as an effective method for determining pollen in the honey sample, and the relative frequency of pollen is used to verify a honey sample in terms of nectar sources (major and minor) [35]. Melissopalynology is the method most capable of discriminating between different types of honey because it determines the pollen sources used by bees to produce honey [34]. El-Sofany et al. [36] argued that in addition to determining the floral and geographical origins of honey, melissopalynological analysis is also useful for the characterization/authentication of honey varieties depending on pollen source of plant species.

Monofloral honey must contain at least 45% of the pollen grains belonging to a single plant species, but this percentage required for classification varies depending on the botanical species [37]. An exception is thyme honey for which Directive 127 of 2004 approved by the Greek Ministry of Agriculture and Food Development states that the product must contain a minimum percentage of 18% pollen grains [38].

In the thyme honey samples from Romania analyzed in this study the percentage of pollen varied between 22–45%. The variation in the percentage of *Rubus* pollen was from 49% to 82.2%. In rape honey, the content of pollen grains varied between 50.12–71% and in mint honey it varied between 46.5–65.02%. The maximum percentage of pollen grains was identified in the case of sunflower honey, respectively 92%.

According to pollen analysis, the honey samples analyzed in this study meet the criterion imposed regarding the percentage of pollen and can be classified as being monofloral: 10 samples of mint, 9 samples of raspberry, 4 samples of thyme, 8 samples of sunflower and 10 samples of rape.

### 3.2. Voltammetric Electronic Tongue

**Figure 1:**
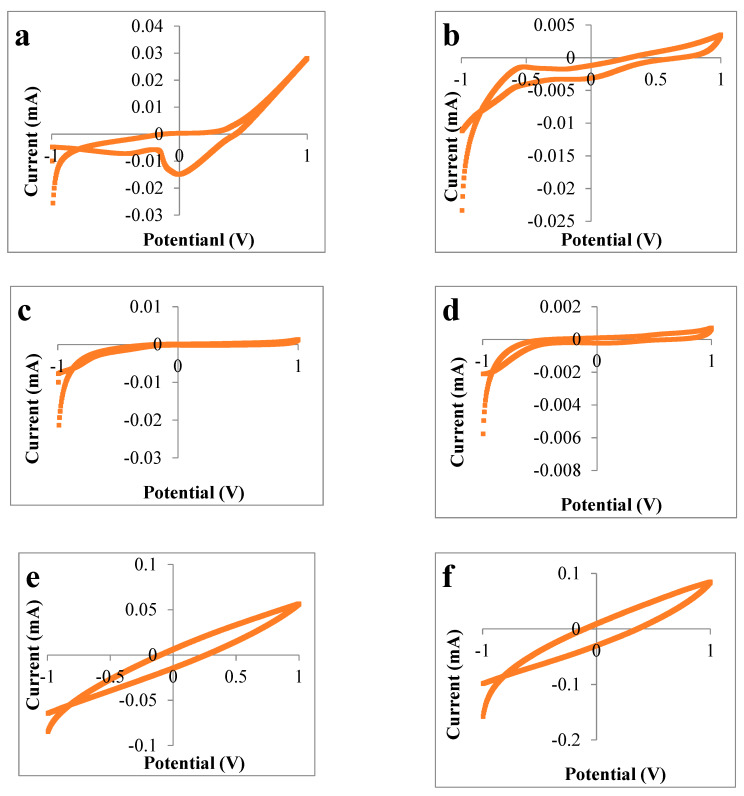
Cyclic voltammograms of rape honey solutions using different electrodes: (**a**) silver electrode, (**b**) platinum electrode, (**c**) gold eletrode, (**d**) glass electrode, (**e**) zinc oxide electrode, (**f**) titanium dioxideelectrode.

Cyclic voltammograms for rape honey are showed in Figure 1 and were obtained with different working electrodes such as Au, Ag, Pt, ZnO, TiO_2_ and a glass electrode. As can be seen from the six cyclic voltammograms, the maximum intensity was measured using titanium dioxide as a working electrode. However, the current varied greatly depending on the working electrodes type, and the smallest intensity was observed for the glass electrode.

Different values of current intensity can be observed depending on the working electrode used; the highest current intensity values were observed in mint honey in the case of the titanium dioxide electrode (0.195 mA). Furthermore, in the case of mint honey, higher values of current intensity for zinc oxide electrode (0.136 mA) and silver electrode (0.109 mA) were also observed. From the 41 honey samples analyzed in this study, in 2 rape honey samples and in 2 raspberry honey samples, higher values of current intensity were recorded with the zinc oxide electrode. In the other 37 honey samples, the maximum value was recorded for the titanium dioxide electrode.

In terms of the value of the current intensity, the titanium dioxide electrode was followed by the zinc oxide and the silver electrode. The lowest values were recorded for the glass electrode for all analyzed samples.

### 3.3. Multivariate Analysis

Using only voltammetric data is very difficult to distinguish the botanical origin of honey samples, and it was necessary to use multivariate analysis techniques such as principal component analysis (PCA) but also linear discriminant analysis (LDA). The maximum currents generated on each electrode and each honey sample were submitted to PCA and LDA. The current of an electrode is not enough for the authentication of a new type; increasing the number of the parameters determined (in our case the current of each electrode) increased the power of an e-tongue for the correct authentication of a honey type.

#### 3.3.1. Principal Component Analysis (PCA)

Principal component analysis (PCA) is a statistical method used to reduce the size of the experimental set of values. This type of statistical analysis is useful for determining how many dimensions are of real importance in interpreting phenomena. The first principal component (PC-1) accounted 85% of the variance, while the second principal component (PC-2) accounted for 9% of the variance; together, they accounted for 94% of initial variability.

**Figure 2:**
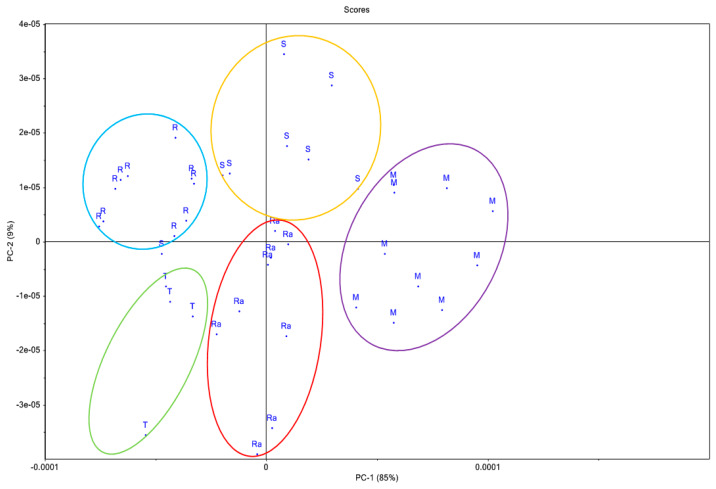
Principal component analysis-scores: RA, raspberry; T, thyme; S, sunflower; M, mint and R, rape honey. Blue ellipse, raspberry honey group; yellow ellipse, sunflower honey group; red ellipse, rape honey group; green ellipse, thyme honey group and purple ellipse, mint honey group.

In Figure 2, the honey types were marked as: RA-raspberry, T-thyme, S-sunflower, M-mint and R-rape honey. The separation of honey samples into groups according to the botanical origin can be observed in Figure 2. The samples of honey of different botanical origin (mint, rape, sunflower, thyme and raspberry) were clearly separated. A sample of raspberry honey might overlap with the sunflower honey ellipse.

**Figure 3:**
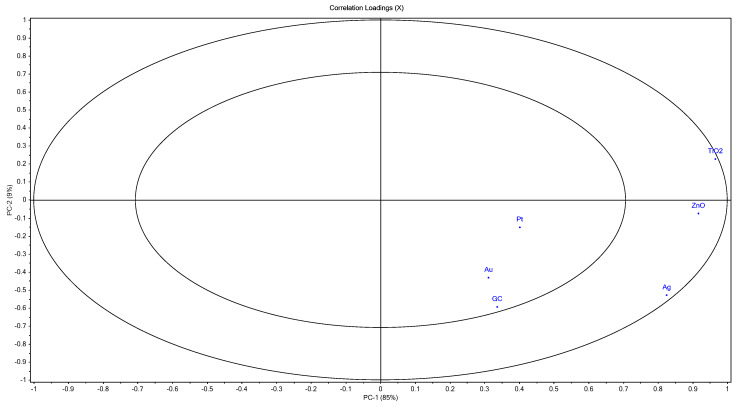
Principal component analysis loadings:Ag, silver electrode; Pt, platinum electrode; Au, gold electrode; GC- glass electrode; ZnO, zinc oxide electrode; TiO_2_, titanium dioxide electrode.

In Figure 3, the parameters used for the projection are abbreviated as: Ag, silver electrode; Pt, platinum electrode; Au, gold electrode; GC, glass electrode; ZnO, zinc oxide electrode; TiO_2_, titanium dioxide electrode. In Figure 3, the parameters in the outer ellipse contribute more than the parameters in the inner ellipse. The small distance between TiO_2_ and ZnO show a strong correlation between variables. The silver electrode and the metal oxide working electrodes in a voltammetric-based electronic tongue can be used for more efficient floral identification with better clustering. Thus, in the case of platinum, gold and glass electrodes, no considerable variations were observed depending on the botanical origin of honey.

#### 3.3.2. Classification of Results with LDA

**Figure 4:**
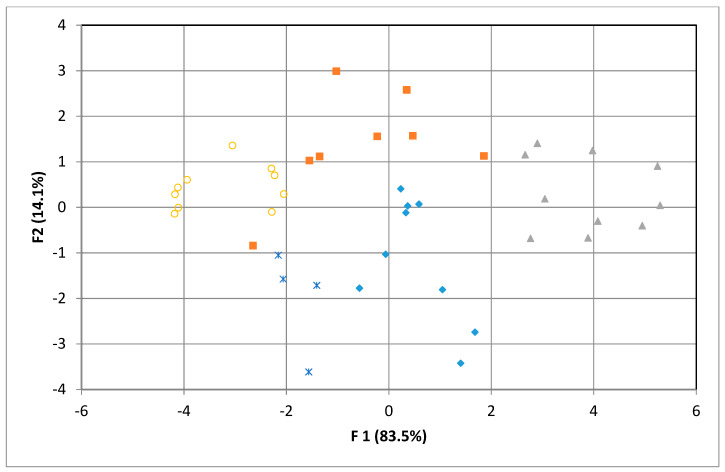
Linear discriminant score plot: blue rhombus, raspberry; red square, sunflower; green triangle, mint; purple circle, rape; blue multiplication sign, thyme.

The classification of honey according to their botanical origin was submitted to LDA based on the maximum potential of each working electrode used. The LDA method used was a stepwise method where every independent variable was entered or removed from the model. The model used the Ag, TiO_2_ and ZnO variables while the Au, Pt and glassy electrode variables were removed. Using this method, it was possible to evaluate the capacity to predict the origin of each honey type correctly. In Table 1, the results of LDA are presented; 92.7% of original grouped cases and 85.4% of cross-validated grouped cases were correctly classified. Rape and mint honeys were 100% correctly classified in original grouped cases and cross-validated grouped cases. The lowest cross-validated grouped cases were observed in the case of raspberry honeys (66.7%). The linear discriminant analysis applied to all the physicochemical parameters resulted in two canonical functions with the eigen values of 7.604 and 1.280 and the values of Wilks’s lambda of 0.042 and 0.358, respectively. Function 1 explained 83.5% of the total variance, while Function 2 explained 14.1%. The highest absolute value, which dominated the first discriminant function, was represented by Ag maximum potential (F1 = 0.741, F2 = −0.358), followed by ZnO maximum potential (F1 = 0.681, F2 = 0.271) and TiO_2_ maximum potential (F1 = 0.662, F2 = 0.624). The second discriminate function was dominated positively by TiO_2_ maximum potential (F1 = 0.662, F2 = 0.624) and negatively by Ag maximum potential (F1 = 0.741, F2 = −0.358). The LDA scores are presented in Figure 4, where the mint, rape and raspberry samples are well defined as groups.

The results obtained in this study were in agreement with those obtained by Sobrino-Gregorio et al. [39] who used the electronic tongue for honey authentication. The voltammetry tests were measured with four working electrodes (Ir, Rh, Pt and Au) housed inside a stainless-steel cylinder used as the body of the electronic tongue. As counting electrode, a circular piece of stainless steel was used, and as reference electrode, a calomel electrode was used. PCA analysis showed that this electronic language system consisting of four metal electrodes (Ir, Rh, Pt, Au) was capable of differentiating pure honey types and pure syrups but also of discriminating honey based on syrups added at different levels. Oroian and Ropciuc [40] analyzed Romanian honey using a e-tongue system consisting of four electrodes (platinum, silver, gold and electrode glass) and reported that platinum and silver electrodes were the most suitable for authenticating honeydew and monofloral honey. Wei et al. [19] reported in their study that the electronic tongue technique was able to classify honey samples from southeast of China (Acacia, Vitex, Astragali, Motherwort, Data, Coptis, Buckwheat and Radix Changll) of different floral and geographical origins. Tiwari et al. [31] achieved with the application of the electronic tongue based on voltammetry and the statistical methods PCA and LDA a successful classification of 80 honey samples from 4 different floral origins.

18 honey samples from different countries (monofloral and polyfloral) and seven types of honey of different botanical origins derived from Morocco were analyzed by Bougrini et al. [41] using voltammetric electronic tongue. In their study, the analysis of the principal components (PCA) showed that the voltammetric electronic tongue system, which was composed of seven voltammetric electrodes, could differentiate the honey samples according to their botanical and geographical origin. El Hassani et al. [42] also reported that they were able to differentiate according to their geographical origin 14 types of Moroccan and French honey using the VE tongue system consisting of seven metal electrodes.

Sousa et al. [43] reached a 100% correct classification of chestnut honey (*Castanea* spp.), lavender (*Lavandula* spp.) and raspberry (*Rubus* spp.) with a potentiometric electronic tongue. The exact classification was obtained after the honey samples were separated according to their color, and then, the authentication of each type of honey was done based on their botanical origin.

Using metallic potentiometric electrodes, which form the electronic tongue system, it is possible to discriminate the floral origin of honey, but it is not possible to differentiate according to the thermal treatment (liquation and pasteurization) applied in the industry [44].

## 4. Conclusions

In this study, it was found that a voltammetric electronic tongue system that used 6 working electrodes (Ag, Au, Pt, ZnO, TiO_2_ and glass electrodes) can be applied in a simple way to authenticate honey samples depending on their botanical origin. Moreover, it was observed that the electrodes of metal oxides, constructed by a simple method, give the best results, demonstrating a good differentiation of the honey samples depending on the floral origin. By comparing these sensors with the Au, Pt and glass electrodes, it was observed that a better differentiation between the five floral types of honey was achieved. The maximum current of each electrode was submitted for PCA and LDA in order to check the suitability of the e-tongue for honey authentication. The results obtained in this study demonstrate the usefulness of cyclic voltammetric tongue for the authentication of honey. Based on the LDA analysis, it was concluded that all samples of rape and mint honey were correctly classified (100%); the raspberry honey samples were classified correctly in a percentage of 66.7%, while the total cross validation correct classification was 85.4%.

## Figures and Tables

**Table 1:** Classification results of the five honeys analyzed using linear discriminant analysis (LDA).

		Honey Type	Predicted Group Membership					Total
			Rape	Sunflower	Mint	Raspberry	Thyme	
Original	Count	Raspberry	9	0	0	0	0	9
		Sunflower	0	6	1	0	1	8
		Mint	0	0	10	0	0	10
		Rape	0	0	0	10	0	10
		Thyme	0	0	0	1	3	4
	%	Raspberry	100	0	0	0	0	100
		Sunflower	0	75	12.5	0	12.5	100
		Mint	0	0	100	0	0	100
		Rape	0	0	0	100	0	100
		Thyme	0	0	0	25	75	100
	Total correct classification							92.7%
Cross validated	Count	Raspberry	6	1	0	0	2	9
		Sunflower	0	6	1	0	1	8
		Mint	0	0	10	0	0	10
		Rape	0	0	0	10	0	10
		Thyme	0	0	0	1	3	4
	%	Raspberry	66.7	11.1	0	0	22.2	100
		Sunflower	0	75.0	12.5	0	12.5	100
		Mint	0	0	100	0	0	100
		Rape	0	0	0	100	0	100
		Thyme	0	0	0	25	75	100
	Total correct classification							85.4%
